# Football Players Do Not Show “Neural Efficiency” in Cortical Activity Related to Visuospatial Information Processing During Football Scenes: An EEG Mapping Study

**DOI:** 10.3389/fpsyg.2019.00890

**Published:** 2019-04-26

**Authors:** Claudio Del Percio, Mauro Franzetti, Adelaide Josy De Matti, Giuseppe Noce, Roberta Lizio, Susanna Lopez, Andrea Soricelli, Raffaele Ferri, Maria Teresa Pascarelli, Marco Rizzo, Antonio Ivano Triggiani, Fabrizio Stocchi, Cristina Limatola, Claudio Babiloni

**Affiliations:** ^1^Department of Physiology and Pharmacology “Vittorio Erspamer”, Sapienza University of Rome, Rome, Italy; ^2^IRCCS SDN, Naples, Italy; ^3^Department of Motor Sciences and Healthiness, University of Naples Parthenope, Naples, Italy; ^4^Oasi Research Institute – IRCCS, Troina, Italy; ^5^Department of Clinical and Experimental Medicine, University of Foggia, Foggia, Italy; ^6^IRCCS San Raffaele Pisana, Rome, Italy; ^7^IRCCS Neuromed, Pozzilli, Italy; ^8^Hospital San Raffaele Cassino, Cassino, Italy

**Keywords:** football (soccer) players, electroencephalography, alpha rhythms, visuospatial information processing, parietal cortex, neural efficiency, situational awareness

## Abstract

This study tested the hypothesis of cortical neural efficiency (i.e., reduced brain activation in experts) in the visuospatial information processing related to football (soccer) scenes in football players. Electroencephalographic data were recorded from 56 scalp electrodes in 13 football players and eight matched non-players during the observation of 70 videos with football actions lasting 2.5 s each. During these videos, the central fixation target changed color from red to blue or vice versa. The videos were watched two times. One time, the subjects were asked to estimate the distance between players during each action (FOOTBALL condition, visuospatial). Another time, they had to estimate if the fixation target was colored for a longer time in red or blue color (CONTROL condition, non-visuospatial). The order of the two conditions was pseudo-randomized across the subjects. Cortical activity was estimated as the percent reduction in power of scalp alpha rhythms (about 8–12 Hz) during the videos compared with a pre-video baseline (event-related desynchronization, ERD). In the FOOTBALL condition, a prominent and bilateral parietal alpha ERD (i.e., cortical activation) was greater in the football players than non-players (*p* < 0.05) in contrast with the neural efficiency hypothesis. In the CONTROL condition, no significant alpha ERD difference was observed. No difference in behavioral response time and accuracy was found between the two groups in any condition. In conclusion, a prominent parietal cortical activity related to visuospatial processes during football scenes was greater in the football players over controls in contrast with the neural efficiency hypothesis.

## Introduction

Previous neuroimaging studies using positron emission tomography (PET), single-photon emission computed tomography (SPECT), and functional magnetic resonance imaging (fMRI) have mapped the cortical activation during cognitive-motor tasks in humans in relation to intelligent quotient (IQ). Compared with subjects having a low IQ, high-IQ people showed weaker cortical frontoparietal activations during the performance of cognitive tasks (Haier et al., 1988, 1992, 2004; Parks et al., 1988; Rypma and D’Esposito, 1999; Rypma et al., 2002, 2005; Ruff et al., 2003). These results support the concept that the most brilliant individuals are characterized by a reduced and circumscribed activation of the cerebral cortex during cognitive tasks, possibly due to the efficiency of neural populations involved in the related information processing. However, this neural efficiency hypothesis does not explain all experimental data, especially when cognitive tasks are challenging. Other neuroimaging evidence pointed to a greater task-related cortical frontoparietal activation in individuals exhibiting a high cognitive performance than those manifesting a low cognitive performance (Gray et al., 2003; Newman et al., 2003).

Single-photon emission computed tomography, PET, and fMRI allow mapping fine topographical details of the cortical neural efficiency in experts, but they could not explore underlying neurophysiological oscillatory mechanisms. These mechanisms can be probed by the analysis of electroencephalographic (EEG) activity. EEG alpha (about 8–12 Hz) rhythms are typically reduced in amplitude, as a sign of cortical activation, during sensory, motor, and cognitive information processing (alpha event-related desynchronization, ERD; Pfurtscheller and Lopes da Silva, 1999; Klimesch, 2012). As a manifestation of cortical neural efficiency, the alpha ERD is less prominent in people with high IQ during several cognitive and working memory tasks (Neubauer et al., 1995, 1999, 2004; Grabner et al., 2004, 2006). Cortical neural efficiency was also evaluated in élite athletes, considered as high-performing people, with puzzling results. Some EEG studies on alpha rhythms have exhibited findings in agreement with the neural efficiency hypothesis. As a matter of fact, the frontoparietal alpha ERD was lower in karate athletes and gymnasts over controls while watching and judging videos reproducing sporting performances (Babiloni et al., 2009, 2010a). Furthermore, the alpha ERD was lower in gun shooters over controls during shooting preparation (Haufler et al., 2000; Janelle et al., 2000; Loze et al., 2001; Del Percio et al., 2009a). Compare to controls, élite karate athletes also exhibited lower alpha ERD during the opening of eyes in the resting state condition (Del Percio et al., 2011), difficult upright standing with eyes closed (Del Percio et al., 2010), simple voluntary hand movements (Del Percio et al., 2010), and a mental arithmetic subtraction task (Duru and Assem, 2018). Furthermore, cyclists with high maximal aerobic power showed lower alpha ERD during cycling compared with control subjects (Ludyga et al., 2016). Finally, tennis table experts over novices presented a lower frontoparietal alpha ERD during the imagery of the response to services represented in videos (Wolf et al., 2014).

Other EEG studies on alpha rhythms have exhibited findings in contrast with the neural efficiency hypothesis. Tennis table experts over novices showed a greater frontal alpha ERD during the imagery of the response to services represented in videos (Wolf et al., 2014). Furthermore, golf experts displayed greater frontal alpha ERD few seconds before successful than unsuccessful putts (Babiloni et al., 2008; Cooke et al., 2014). They also exhibited greater frontal alpha ERD compared with novices (Cooke et al., 2014). In the same line, the frontal alpha ERD was greater after the unsuccessful putts in the golf experts over the novices, as expected when substantial cognitive sources are allocated for correcting performance parameters in experts (Cooke et al., 2015). Finally, frontal alpha ERD was greater in karate and fencing athletes over controls who were maintaining the equilibrium using visual information about the surrounding environment (Del Percio et al., 2007).

The present study tested the hypothesis of experts’ cortical neural efficiency in the visuospatial information processing during the observation of football (soccer) scenes. For this purpose, the cortical activation was indexed by the alpha ERD computed in football (soccer) players and control subjects during videos of football actions requiring visuospatial demands. The same videos were used for a control condition focused on visual non-spatial demands.

## Materials and Methods

### Participants

Thirteen non-professional male football (soccer) players and eight age- and sex-matched control non-players were enrolled in this experiment. All subjects were right-handed as measured by the Edinburgh Inventory (players: mean of 0.4; non-players: 0.6) and gave their written and informed consent under the World Medical Association’s Declaration of Helsinki. They were free to withdraw from the study at any time they wanted. The present study and protocol were reviewed and approved by the Ethics Committee of the Department of Physiology and Pharmacology “Vittorio Erspamer”, Sapienza University of Rome.

The football players have been practicing football at the agonistic level (i.e., regular annual tournament at regional or national level) for at least 10 years (mean of 12.7 years ± 0.3 standard error, SE) and a minimum of 4 times a week during regular sporting seasons except for the event of sporadic sport accidents or diseases. The players’ average age was of 25.1 years (±0.3 standard error, SE).

In the preliminary interviews, the control subjects reported that they had neither practiced football at the agonistic level nor had participated at official amateur football tournaments. They have been occasionally playing football (i.e., on average, once a month) with relatives and friends, especially at the time of high school. The average age of the control subjects was of 25.6 years (±0.2 SE).

### Experimental Procedure

Electroencephalography data were recorded during the observation of 70 videos of football actions lasting 2.5 s each. The videos were presented on a computer monitor placed about 1 m in front of the subject receiving the EEG recording.

The subject kept the left index (non-dominant hand) on a key placed at the left-down angle of the computer keyboard (e.g., Ctrl) while the right index (dominant hand) was on a key placed at its right-down angle (e.g., enter key).

**Figure 1:**
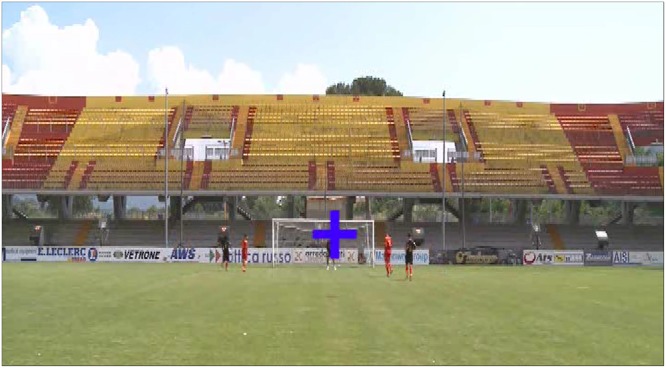
Electroencephalography (EEG) data were recorded during the observation of 70 videos of football actions lasting 2.5 s each on a computer monitor placed in front of the subject receiving the EEG recording. The subject kept the left index (non-dominant hand) on a key placed at the left-down angle of the computer keyboard (e.g., Ctrl) while the right index (dominant hand) was on a key placed at its right-down angle (e.g., enter key). In each video, two forwards were running on the goal (one on the left and the other on the right of a fixation cross at the center of the monitor). Meanwhile, two defenders were running near them to control their movements. During the videos, a central fixation cross changed color either from red to blue or from blue to red. At the end of the video, the subject had to press one of the two mentioned keys of the keyboard within a maximum response time of 5 s. For a description of the cognitive task planned in the EEG experiment, please see Section “Materials and Methods”.

Each video (Figure 1) represented a paradigmatic football action planned by a professional football coach. In each video, two forwards were running on the goal (one on the left and the other on the right of a fixation cross at the center of the monitor). Meanwhile, two defenders were running near them to control their movements. During the videos, a central fixation cross changed color either from red to blue or from blue to red. The respective permanence time of the red and blue colors in the cross changed video-by-video in a pseudorandom and matched order. No video showed red and blue colors in the cross for an equal time. At the end of the video, the subject had to press one of the two mentioned keys of the keyboard within a maximum response time of 5 s.

During the EEG recordings, the 70 videos were watched two times. One time, the subject was asked to estimate the distance between the players during the action (FOOTBALL condition). Another time, they were asked to estimate if the central fixation cross was colored for a longer time in red or blue (CONTROL condition). The order of the two conditions was pseudo-randomized across subjects.

In the FOOTBALL condition, the subject had to press the mentioned left key if he thought that there was globally more distance between the forward and the defender at the left side of the monitor compared with the distance between the forward and the defender at the right side. He was instructed to estimate mentally the average distance between the two couples of players (i.e., one forward and one defender) across the whole action of 2.5 s. Vice versa, the subject had to press the right key if he perceived more distance between the forward and the defender at the right side of the monitor compared with the distance between the forward and the defender at the right side.

In the CONTROL condition, the subject had to press the mentioned left key if he thought that there was globally a longer permanence of the red over blue in the central fixation cross. Vice versa, he had to press the right key for a longer permanence of the blue over red in the central fixation cross.

### Settings of the EEG Recordings

The EEG data were recorded continuously from 56 monopolar exploring electrodes (bandpass: 0.01–100 Hz; sampling rate: 512 Hz; EB Neuro-Be-plus©, Florence, Italy) positioned on the scalp according to the 10–10 system. The electrical reference was located between the AFz and Fz electrodes and the ground was placed between the Pz and Oz electrodes. The impedance of all exploring electrodes was kept below 5 kΩ. Simultaneously, vertical and horizontal electro-oculographic (EOG) data were registered to monitor blinks and saccadic eye movements (bandpass: 0.1–100 Hz; sampling rate: 512 Hz).

### Preliminary Data Analysis

The EEG data related to the 70 videos were divided into 70 epochs having a duration of 8 s each. Any epoch spanned from -4 to +4 s with reference to the beginning of the video as a zero time. The EEG epochs were controlled for instrumental, blinking, ocular, and muscle artifacts. A home-made software implementing an auto-regressive procedure (Moretti et al., 2003) corrected blinking and ocular artifacts. Two EEG experts (CDP and AJDM) controlled and confirmed manually the automatic selection and correction of the procedure for all EEG epochs, with special attention to the residual contamination of the EEG signal due to head displacements, facial and neck muscle tension, blinks, and saccadic eye movements. Artifact-free EEG epochs were considered for the following analysis.

### Analysis of EEG Alpha Rhythms

Spectral analysis of the artifact-free EEG epochs was based on the computation of Fast Fourier Transform (FFT) approach using the Welch technique and the Hanning function (frequency of 1 Hz resolution). The outcome was the estimation of the EEG power density at any frequency from 1 to 45 Hz.

According to a previous study of our group (Moretti et al., 2004), the frequency bands of interest in the alpha range were individually identified based on the individual alpha frequency peak (IAF). In the EEG power density spectrum, the IAF was defined as the maximum power density peak between 6 and 14 Hz. These frequency landmarks were previously well described by Klimesch (1999, 1996) and Klimesch et al. (1998).

The IAF was individually computed for each subject involved in the study. Based on the IAF, we estimated the low-frequency alpha band from IAF-2 Hz to IAF while the high-frequency alpha band ranged from IAF to IAF+2 Hz. On average, we found that IAF was 10.6 Hz (±0.3 SE) in the football players and 9.8 Hz (±0.4 SE) in the control non-players. There was no statistically significant difference between the two groups in the IAF as assessed by a *t*-test (*p* > 0.05).

### Computation of Alpha Event-Related Desynchronization/Synchronization (ERD/ERS)

The alpha ERD/ERS was defined as the decrease/increase in the percentage of the alpha power density during the video compared with a period lasting 1 s immediately preceding its beginning, from –1 s to the zerotime (i.e., the beginning of the video). Specifically, two periods of the video were of interest: T1 from +0.5 to +1.5 s and T2 from +1.5 to +2.5 s. Practically, the ERD% was calculated by the following formula: (video – pre-video)/pre-video × 100 (Pfurtscheller and Lopes da Silva, 1999). As the outcome of the formula, negative percentage values represented the alpha ERD as a sign of cortical activation while the positive percentage values represented the alpha ERS as a sign of cortical deactivation. In this formula, the “video” indicates the alpha power density at T1 or T2 and the “pre-video” denotes the alpha power density during the period lasting 1 s immediately preceding the video beginning. Of note, the alpha ERD calculation was performed for both alpha sub-bands, namely the low- and high-frequency alpha sub-bands.

### Topographic Mapping of the Alpha ERD/ERS

A spline interpolating function (Babiloni et al., 1996) was used to compute topographic maps (256 hues) of alpha ERD/ERS values at 56 electrode sites of augmented 10–20 system. This procedure has been successfully used to compute topographic maps of alpha ERD/ERS in our previous studies in élite fencing and karate athletes (Del Percio et al., 2007, 2008, 2009a, 2010). Noteworthy, the present procedure has some important advantages and a minor disadvantage. It interpolates alpha ERD/ERS values exactly at the same scalp sites in all experimental subjects, thus overcoming the spatial errors due to the individual shift of the positioning of the electrode cap across subjects. In this procedure, interpolated alpha ERD/ERS values were projected over a 3-D “quasi-realistic” template model of cerebral cortex. This template model was constructed based on the magnetic resonance data of 152 subjects, digitized at Brain Imaging Center of the Montreal Neurological Institute (SPM96)^1^. It is commonly considered as an acceptable template for the rendering of group neuroimaging data. As a disadvantage of the present approach, spline functions might introduce some minor estimation errors at the border electrodes of the montage. For this reason, we did not consider alpha ERD/ERS solutions in the border regions of the electrode montage.

### Statistical Analysis

Statistical comparisons were performed by the analysis of variance (ANOVA). With the ANOVA analysis, the Mauchly test evaluated the sphericity assumption, when necessary. Correction of the degrees of freedom was made by the Greenhouse–Geisser procedure, while the Duncan test was used for the *post hoc* analysis (*p* < 0.05).

In the first statistical session (behavioral data), we tested the hypothesis of higher accuracy (i.e., percentage of correct responses) and shorter reaction time in behavioral responses of the football players compared with the control non-players in the FOOTBALL but not the CONTROL condition (*p* < 0.05). This hypothesis was evaluated by an ANOVA having the response accuracy as a dependent variable and Group (football players, non-players; independent variable) and Condition (football, control) as factors. Similarly, another ANOVA used the reaction time as a dependent variable and Group (football players, non-players; independent variable) and Condition (football, control) as factors.

In the second statistical session (EEG data), we tested the hypothesis of mean differences in the alpha ERD between the groups of football players and control non-players in the FOOTBALL but not the CONTROL condition (*p* < 0.05). This hypothesis was evaluated by two ANOVAs, one for the low-frequency alpha band and the other for the high-frequency alpha band. These ANOVAs had the alpha ERD as a dependent variable and Group (football players, non-players; independent variable), Electrode (F3, Fz, F4, C3, Cz, C4, P3, Pz, P4, O1, O2), and Condition (football, control) as factors. The results of this statistical analysis were controlled by the Grubbs test (*p* < 0.001) for the presence of outliers.

## Results

### Behavioral Data

**Figure 2:**
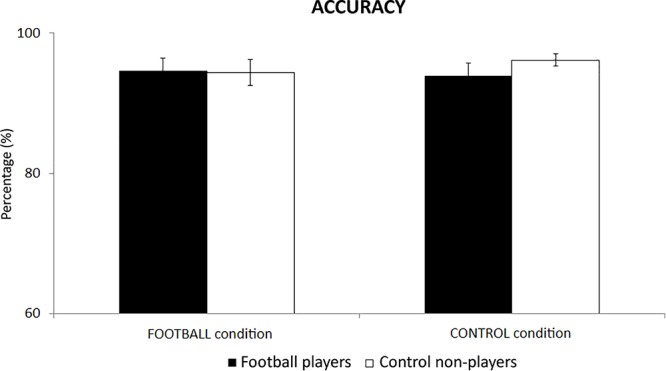
The mean accuracy of behavioral responses in the football players and control non-players in the two cognitive tasks of the present experiments, namely the FOOTBALL and the CONTROL condition.

In the football players, the mean accuracy of behavioral responses was of 95% (±1.9 SE) in the FOOTBALL condition and 94% (±1.8 SE) in the CONTROL condition. Similarly, high levels of the mean accuracy were observed in the control non-players. Their mean accuracy was of 94% (±1.9 SE) in the FOOTBALL condition and 96% (±0.9 SE) in the CONTROL condition. Figure 2 plots these values for illustrative purposes. The ANOVA showed no statistically significant differences between the two groups or between the conditions (*p* > 0.05).

**Figure 3:**
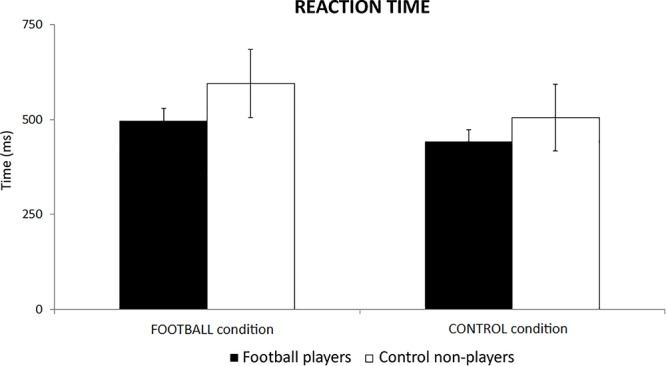
The mean reaction time of behavioral responses in the football players and control non-players in the two cognitive tasks of the present experiments, namely the FOOTBALL and the CONTROL condition.

In the football players, the mean reaction time of behavioral responses was of 496 ms (±33 SE) in the FOOTBALL condition and 441 ms (±32 SE) in the CONTROL condition. In the control non-players, the mean reaction time was of 594 ms (±90 SE) in the FOOTBALL condition and 505 ms (±88 SE) in the CONTROL condition. Figure 3 plots these values for illustrative purposes. The ANOVA showed no statistically significant effect (*p* > 0.05), despite some differences in the mean reaction time between the two groups.

### EEG Data: Alpha ERD/ERS

**Figure 4:**
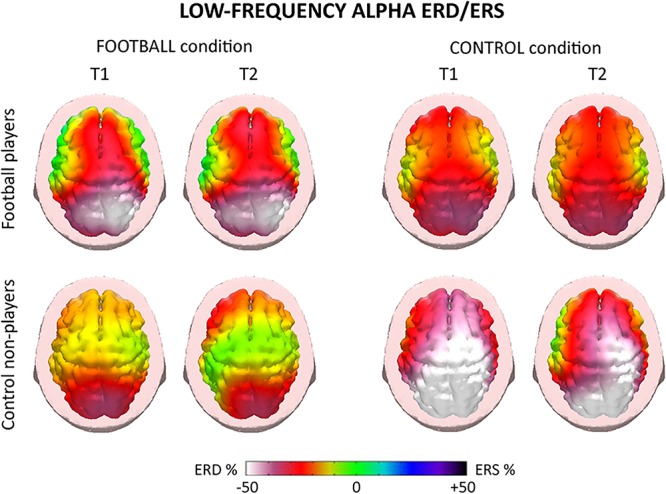
Topographic maps of the alpha event-related desynchronization/synchronization (ERD/ERS) in the football players and control non-players for the “FOOTBALL” and the “CONTROL” condition of the present EEG experiments. These maps refer to low-frequency alpha rhythms (about 8–10 Hz) measured during the videos of 2.5 s showing football actions. Two periods of the videos were of interest, namely T1 and T2. T1 corresponded to the period from +0.5 to +1.5 s, while T2 corresponded to the period from +1.5 to +2.5 s, using the starting of the video as a zerotime. The alpha ERD/ERS during the T1 and T2 periods were computed with reference to a pre-video period from –1.0 s to the zerotime. Color scale: maximum percentage values of the ERD (i.e., cortical activation) and ERS (i.e., cortical deactivation) are represented in white and purple, respectively. The color palette reports the reference ERD/ERS percentage values.

For illustrative purposes, Figure 4, 5 plot the topographic maps of the alpha ERD/ERS in the football players and control non-players for the FOOTBALL and the CONTROL condition. Quite similar ERD spatial distributions for low- and high-frequency alpha sub-bands were observed, so a general description valid for both alpha sub-bands follows. In the FOOTBALL condition, the topographic maps in both football players and control non-players were characterized by alpha ERD values prominent in bilateral parietal areas during the two periods of interest (i.e., T1 and T2), in line with the visuospatial nature of the task. Compared with the control non-players, the football players showed greater alpha ERD values. Of note, the CONTROL condition did not present the above differences between the two groups. Furthermore, there was no remarkable prominence of the alpha ERD in parietal areas, compatible with the non-spatial nature of the task. Finally, the control non-players exhibited a diffuse and slightly greater alpha ERD compared with the football players.

**Figure 5:**
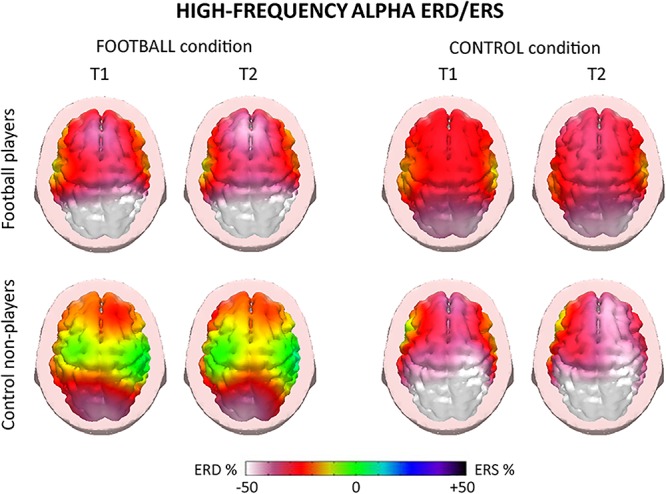
Topographic maps of the high-frequency (about 10–12 Hz) alpha ERD/ERS in the football players and control non-players for the “FOOTBALL” and the “CONTROL” condition. These maps refer to the two periods of the videos considered, namely T1 and T2. Color scale: maximum percentage values of the ERD (i.e., cortical activation) and ERS (i.e., cortical deactivation) are represented in white and purple, respectively. The color palette reports the reference ERD/ERS percentage values.

**Figure 6:**
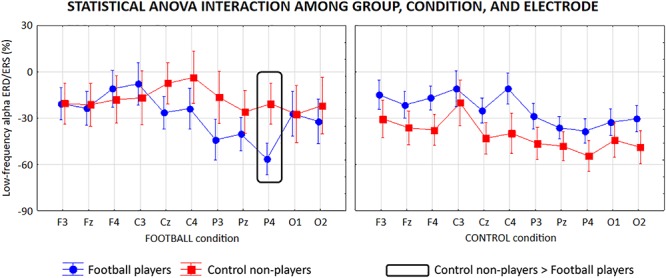
Mean values (±SE) of the low-frequency (about 8–10 Hz) alpha ERD/ERS relative to a statistical ANOVA interaction among the factors Group (football players, control non-players; independent variable), Condition (FOOTBALL, CONTROL), and Electrode (F3, Fz, F4, C3, Cz, C4, P3, Pz, P4, O1, O2). Legend: the rectangles indicate the electrodes in which the low-frequency alpha ERD/ERS values showed statistically significant differences between the two groups in the FOOTBALL condition (Duncan *post hoc* test, *p* < 0.05).

For the low-frequency alpha sub-band, the ANOVA showed a statistical interaction (*F* = 3.1; *p* < 0.001) among the factors Group (football players, control non-players; independent variable), Condition (FOOTBALL, CONTROL), and Electrode (F3, Fz, F4, C3, Cz, C4, P3, Pz, P4, O1, O2; Figure 6). The Duncan *post hoc* test unveiled that compared with the control non-players, the football players showed a greater alpha ERD at P4 (*p* = 0.01) electrode in the FOOTBALL condition, in contrast with the neural efficiency hypothesis. In the CONTROL condition, no difference in the alpha ERD was observed between the two groups (*p* > 0.05), despite some differences in the mean values.

**Figure 7:**
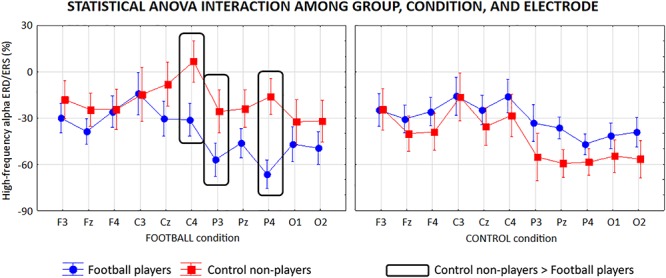
Mean values (±SE) of the high-frequency (about 8–10 Hz) alpha ERD/ERS relative to a statistical ANOVA interaction among the factors Group (football players, control non-players; independent variable), Condition (FOOTBALL, CONTROL), and Electrode (F3, Fz, F4, C3, Cz, C4, P3, Pz, P4, O1, O2). Legend: the rectangles indicate the electrodes in which the high-frequency alpha ERD/ERS values showed statistically significant differences between the two groups in the FOOTBALL condition (Duncan *post hoc* test, *p* < 0.05).

The high-frequency alpha sub-band presented similar ANOVA results reported for the low-frequency alpha sub-band. Specifically, the ANOVA showed a statistical interaction (*F* = 4.6, *p* < 0.0001) among the factors Group (football players, control non-players; independent variable), Condition (FOOTBALL, CONTROL), and Electrode (F3, Fz, F4, C3, Cz, C4, P3, Pz, P4, O1, O2; Figure 7). Furthermore, the Duncan *post hoc* test showed that compared with the control non-players, the football players showed a greater alpha ERD at C4 (*p* = 0.01), P3 (*p* = 0.04), and P4 (*p* = 0.002) electrodes in the FOOTBALL condition (*p* > 0.05).

Of note, the above findings were not due to outliers from those individual low- and high-frequency alpha ERD/ERS values, as shown by Grubbs’ test with an arbitrary threshold of *p* < 0.001.

### Control Analysis

A control correlation analysis was performed to evaluate the relationships between the behavioral performance and the alpha ERD (cortical activation) related to visuospatial processes during the FOOTBALL condition. To address this issue, Pearson test (*p* < 0.05) computed the linear correlation between the accuracy and reaction time of behavioral responses vs. the alpha ERD during that condition. For this purpose, we only considered the low- and high-frequency alpha ERD solutions showing statistically significant differences (*p* < 0.05) between football players and control subjects in the FOOTBALL condition (i.e., low-frequency alpha ERD at P4 electrode and high-frequency alpha ERD at C4, P3, and P4 electrodes). Specifically, the correlation analysis was performed in two sessions. In the first session, football players and control non-players were considered as whole a group. In the second session, any single group was considered separately. No statistically significant effect was observed (*p* > 0.05).

## Discussion

In the present study, we probed the hypothesis of cortical neural efficiency in football (soccer) players involved in the processing of visuospatial information during videos with football actions. In the FOOTBALL condition, the task required the analysis of the relative distance between forwards and defenders during attacks on goal. The alpha ERD was used as an index of the cortical neural efficiency.

### Cortical Activity of Football Players Does Not Fit Neural Efficiency Mode in Visuospatial Information Processing During Football Scenes

The main results showed that a large-band alpha ERD (about 8–12 Hz) was prominent in bilateral parietal areas in the football players over control non-players during the FOOTBALL condition, in contrast with the cortical neural efficiency hypothesis. Of note, compared with the control non-players, the football players showed a reduced reaction time (<100 ms) in the FOOTBALL condition, but this difference was not statistically significant (*p* > 0.05). In the preliminary interviews, the control non-players reported that they have been occasionally playing football (i.e., about once a month), so they are not “naïve” about the game (In Italy, it is rare to find young adults who do not play this sport at all). However, we think that the mentioned lack of differences in behavioral performances was not mainly due to such minor practice of football in the control non-players. Rather, it may be due to a “floor” effect of the task, due to the lack of specific psychomotor demands trained in football players. In other words, the present computerized visuospatial task of the FOOTBALL condition did not require the combination of visuospatial and psychomotor skills typically trained in football practice. Furthermore, the lack of differences in behavioral performances in the mentioned cognitive task may be considered as an advantage for the evaluation of the neural efficiency hypothesis. Indeed, this hypothesis stands on the principle that compared with learners’ brain, that of experts is trained to reach the required level of performance with a selective cerebral activation and the inhibition of irrelevant neural circuits. In this line, the comparison of brain activity can successfully test the neural efficiency hypothesis even better when task performance is paired in learners and experts, as eventual performance differences between groups may be considered as a confounding variable in the analysis of alpha ERD/ERS. In previous studies carried out in élite karate and fencing athletes, no behavioral difference was found between groups of athletes and controls in an equilibrium task (Del Percio et al., 2007, 2009b). That finding allowed us to interpret differences in EEG alpha rhythms as a possible effect of the neural efficiency (Del Percio et al., 2007, 2009b).

The main EEG results of the present study may not be explained by parallel visual non-spatial and short-term memory processes. Indeed, the CONTROL condition used the same videos with a different request to the subjects. They had to estimate the temporal permanence of the red and blue colors in the central fixation cross of the videos. In that CONTROL condition, no substantial difference in the alpha ERD was observed between the two groups.

The EEG results of the present study complement and extend previous evidence challenging the hypothesis of a cortical neural efficiency in athletes engaged in visuospatial tasks. Indeed, a previous fMRI study has shown that the parietal-occipital activation was greater in professional rugby players over beginners engaged in a task of mental spatial rotation of complex objects (Sekiguchi et al., 2011). In another study, the parietal alpha ERD (i.e., cortical activation) was greater in élite karate and fencing athletes over non-athletes during the upright balance grounded on the environmental visuospatial information (Del Percio et al., 2007). Furthermore, the frontal alpha ERD in élite golf athletes was greater in successful than unsuccessful putts, namely a performance implying a complex integration of visuospatial information and multi-joint body movements (Babiloni et al., 2008). On the other hand, the present results are at odds with previous alpha ERD studies leading support to the hypothesis of a cortical neural efficiency in athletes. In those studies, the frontoparietal alpha ERD was lower in elite karate and fencing athletes over control subjects during the observation and global judgment of athletes’ performances in videos (Babiloni et al., 2009, 2010b). Furthermore, the alpha ERD was lower in gun shooters over control subjects during actual pistol shooting performances, a task implying a visuospatial information processing in conjunction with a static control of the posture (Haufler et al., 2000; Janelle et al., 2000; Loze et al., 2001; Del Percio et al., 2009b).

### Neurophysiological Oscillatory Mechanisms Underpinning Visuospatial Information Processing in Football Players

The present alpha ERD results may be explained by neurophysiological oscillatory mechanisms underpinning visuospatial information processing in parietal areas. In wakefulness, cortical pyramidal neurons in parietal areas may generate ample posterior alpha rhythms due to synchronizing oscillatory signals (8–12 Hz) within a feedback loop spanning cortical, basal ganglia, and thalamic neurons (Pfurtscheller and Lopes da Silva, 1999; Hughes et al., 2008; Lörincz et al., 2008; Klimesch, 2012). That synchronizing mechanism may inhibit visuospatial and sensorimotor information flows toward and from the parietal cerebral cortex, while the opposite desynchronizing mechanism may dis-inhibit that information flow (Pfurtscheller and Lopes da Silva, 1999; Lörincz et al., 2008; Klimesch, 2012). In the present FOOTBALL condition, visuospatial stimuli (i.e., changing trajectories and relative distances between forwards and defenders) may trigger a mechanism of desynchronization of those neurons generating the observed parietal alpha ERD. Overall, this neurophysiological mechanism may release the mentioned background inhibition in the transmission and retrieval of relevant sensorimotor and visuospatial representations in parietal neural networks (Steriade and Llinas, 1988; Brunia, 1999; Pfurtscheller and Lopes da Silva, 1999; Deeny et al., 2003; Hughes et al., 2008; Lörincz et al., 2008; Klimesch, 2012).

Why did previous EEG studies in experts report contrasting results about the cortical neural efficiency in visuospatial tasks? How can or cannot the neural efficiency mode take place in experts? Here we can speculate about these issues. Cortical neural efficiency in experts might result from a long training with repeated episodes and associations among relevant stimuli, behavioral responses, and rewards. Those associations may consolidate synaptic connections into selective brain neural networks impinging on the cerebral cortex. During new episodes, neurophysiological oscillatory mechanisms (e.g., alpha ERD, and gamma ERS > 30 Hz) may selectively re-activate relevant synapses/networks and inhibit the irrelevant ones, thus establishing a cortical neural efficiency (Babiloni et al., 2004, 2006; Murakami et al., 2008). In experts, the neural efficiency mode may be formed by relatively few variants of those associations across episodes and may sub-serve relatively simple and automatic visuospatial information processes as those related to static pistol shooting performances and a global judgment of sporting actions (Haufler et al., 2000; Janelle et al., 2000; Loze et al., 2001; Babiloni et al., 2009, 2010a; Del Percio et al., 2009b).

Keeping in mind the above considerations, it can be speculated that football actions may be intrinsically too unpredictable and variable to be associated with encoding, retrieval, and automatic response processes encrypted within very circumscribed brain neural networks in experts. As a result, the representation of those processes in extended brain networks may make their visuospatial information processing not in line with a neural efficiency mode. Furthermore, those extended brain networks may make visuospatial “situational awareness,” decision-making processes, and responses more flexible (Gabbett et al., 2008; Roca et al., 2013; Di Tore et al., 2018). This speculative model may explain what happens in athletes when brain information processing of sporting scenes is challenging, requiring intense involvement of cognitive representations in associative temporal, parietal, and occipital neural networks. This intense involvement may be required for visuospatial information processing and decision-making in some situational sports such as football (soccer), tennis, rugby, basket, handball volleyball, and hockey (Williams et al., 1994; Williams and Davids, 1998; Williams, 2000). In those sports, teammates and opponents may emit partially unpredictable behaviors, cause unexpected ball trajectories, and require quick decisions and adaptive behavioral responses.

## Conclusion

This study tested the hypothesis of athletes’ cortical neural efficiency in the visuospatial information processing related to the observation of football scenes. In the FOOTBALL (visuospatial) condition, prominent and bilateral parietal alpha ERD (i.e., cortical activation) was significantly greater in the football players than non-players. In contrast, no significant ERD differences were observed in the CONTROL (non-visuospatial) condition. These results suggest that the parietal cortical activity of football players did not show a neural efficiency functioning during the complex visuospatial information processing related to football attacks on goal. Future studies will have to test the hypothesis of greater parietal alpha ERD and more accurate behavioral responses in football players over non-players with highly challenging football attacks on goal. Furthermore, future studies will have to measure “situational awareness” with psychometric tests and enrich the neurophysiological model with the analysis of alpha functional connectivity (i.e., coherence), which was proved to be a very fruitful approach to unveil informative neural correlates of mental processes in athletes during sporting performances (Zhu et al., 2011; Gallicchio et al., 2016).

## Ethics Statement

All subjects gave their written and informed consent under the World Medical Association’s Declaration of Helsinki. They were free to withdraw from the study at any time they wanted. The present study and protocol were reviewed and approved by the Ethics Committee of the Department of Physiology and Pharmacology “Vittorio Erspamer”, Sapienza University of Rome.

## Author Contributions

CDP and CB involved substantial contributions to the design of the project idea and wrote the article. MF contributed to the project idea and subjects selection. ADM contributed to the subjects selection and handling. GN, RL, and SL performed EEG data recording and analyses, and wrote the article. AS, RF, FS, and CL involved critical revision for important intellectual content. MP, MR, and AT performed data analyses.

## Conflict of Interest Statement

The authors declare that the research was conducted in the absence of any commercial or financial relationships that could be construed as a potential conflict of interest.

## Footnotes

1 www.mni.mcgill.ca
