# Corrigendum: Football Players Do Not Show “Neural Efficiency” in Cortical Activity Related to Visuospatial Information Processing During Football Scenes: An EEG Mapping Study

**DOI:** 10.3389/fpsyg.2019.01877

**Published:** 2019-08-27

**Authors:** Claudio Del Percio, Mauro Franzetti, Adelaide Josy De Matti, Giuseppe Noce, Roberta Lizio, Susanna Lopez, Andrea Soricelli, Raffaele Ferri, Maria Teresa Pascarelli, Marco Rizzo, Antonio Ivano Triggiani, Fabrizio Stocchi, Cristina Limatola, Claudio Babiloni

**Affiliations:** ^1^Department of Physiology and Pharmacology “Vittorio Erspamer”, Sapienza University of Rome, Rome, Italy; ^2^IRCCS SDN, Naples, Italy; ^3^Department of Motor Sciences and Healthiness, University of Naples Parthenope, Naples, Italy; ^4^Oasi Research Institute – IRCCS, Troina, Italy; ^5^Department of Clinical and Experimental Medicine, University of Foggia, Foggia, Italy; ^6^IRCCS San Raffaele Pisana, Rome, Italy; ^7^IRCCS Neuromed, Pozzilli, Italy; ^8^Hospital San Raffaele Cassino, Cassino, Italy

**Keywords:** football (soccer) players, electroencephalography, alpha rhythms, visuospatial information processing, parietal cortex, neural efficiency, situational awareness

In the published article, there were errors regarding the affiliations for Dr. Roberta Lizio and Prof. Cristina Limatola. The correct affiliation for Dr. Roberta Lizio is ^2^IRCCS SDN, Naples, Italy. Furthermore, as well as having affiliation ^1^Department of Physiology and Pharmacology “Vittorio Erspamer”, Sapienza University of Rome, Rome, Italy, Prof. Cristina Limatola should also have ^7^IRCCS Neuromed, Pozzilli, Italy.

The authors apologize for these errors and state that these do not change the scientific conclusions of the article in any way. The original article has been updated.

